# Low body surface area predicts hepatotoxicity of nintedanib in patients with idiopathic pulmonary fibrosis

**DOI:** 10.1038/s41598-017-11321-x

**Published:** 2017-09-07

**Authors:** Satoshi Ikeda, Akimasa Sekine, Tomohisa Baba, Yumie Yamanaka, Shinko Sadoyama, Hideaki Yamakawa, Tsuneyuki Oda, Ryo Okuda, Hideya Kitamura, Koji Okudela, Tae Iwasawa, Kenichi Ohashi, Tamiko Takemura, Takashi Ogura

**Affiliations:** 1 Kanagawa Cardiovascular and Respiratory Center, Department of Respiratory Medicine, Yokohama, 236-0051 Japan; 2 Yokohama-city University Graduate School of Medicine, Department of Pathology, Yokohama, 236-0004 Japan; 3 Kanagawa Cardiovascular and Respiratory Center, Department of Radiology, Yokohama, 236-0051 Japan; 4 Japanese Red Cross Medical Center, Department of Pathology, Tokyo, 150-8935 Japan

## Abstract

After the commercialization of nintedanib in Japan, a high incidence of hepatotoxicity resulting in treatment interruption was noted in idiopathic pulmonary fibrosis (IPF) patients treated with nintedanib in our hospital. This study aimed to clarify the risk factors for hepatotoxicity of nintedanib. Sixty-eight consecutive cases of IPF newly treated with nintedanib at a dose of 150 mg twice daily from September 2015 to September 2016 were enrolled: 46 patients (67.6%) exhibited aspartate aminotransferase (AST) and/or alanine aminotransferase (ALT) elevation and 16 patients (23.5%) also had a Common Terminology Criteria for Adverse Events (CTCAE) grade ≥2. Body surface area (BSA) was significantly lower in the CTCAE grade ≥2 group than in another group. A multivariate logistic regression analysis showed that the association between BSA and AST/ALT elevation with CTCAE grade ≥2 was statistically significant. Eight of 10 patients who resumed nintedanib at a reduced dose of 100 mg twice daily after interruption due to hepatotoxicity did not again develop AST/ALT elevation. In conclusion, a low BSA was associated with hepatotoxicity of nintedanib at a dose of 150 mg twice daily. It would be a good option for patients with a small physique to start nintedanib at a dose of 100 mg twice daily and then increase if possible after confirming its safety.

## Introduction

Nintedanib is a small-molecule tyrosine kinase inhibitor that inhibits vascular endothelial growth factor, platelet-derived growth factor, and fibroblast growth factor^1, 2^. Two randomized phase III trials (INPULSIS™-1 and -2) showed that nintedanib reduced the decline in forced vital capacity (FVC) in patients with idiopathic pulmonary fibrosis (IPF) with a manageable side effect profile^3–5^. Based on these trial results, nintedanib was approved for IPF by the Pharmaceutical and Medical Devices Agency in Japan and was clinically deployed from September 2015.

However, after the commercialization of nintedanib for IPF, a high incidence of hepatotoxicity resulting in treatment interruption was noted in our hospital. We previously reported that 11 of 32 patients with IPF newly treated with nintedanib at a dose of 150 mg twice daily from September to December 2015 exhibited aspartate aminotransferase (AST) and/or alanine aminotransferase (ALT) elevation with the Common Terminology Criteria for Adverse Events (CTCAE) grade ≥2^6, 7^. In addition, this study showed that body mass index (BMI) and body surface area (BSA) were significantly lower in patients who presented with hepatotoxicity, and that hepatotoxicity was more frequent when BSA was <1.50 m^2^ and BMI was <22.

In the present study, we accumulated more cases and conducted further investigations to clarify the risk factors for hepatotoxicity of nintedanib in patients with IPF.

## Results

### Characteristics

Patient characteristics are summarized in Table 1. Sixty-eight patients with IPF were enrolled in this study of which 76.5% were male with a median age of 72 years. The median body weight, BMI, and BSA estimated using the Du Bois formula were 57.7 kg, 22.1, and 1.64 m^2^, respectively. The median % FVC and % diffusing capacity for lung carbon monoxide (DLCO) at baseline were 62.3% and 50.8%, respectively. The most common concomitant drug was prednisolone (16.2%) followed by tacrolimus (7.4%). The median follow up period was 235 days (data cut-off date was October 19, 2016).

**Table 1:** Patient characteristics.

	Our patients	Nintedanib group in INPULSIS trials	
		Japanese	Overall
**Number of patients**	68	76	638
**Age**	72.0 (68.0–76.0)	68.4 ± 7.6*	66.6 ± 8.1*
**Gender (male/female)**	52/16	62/14	507/131
Physique			
Height (cm)	164 (158–169)	—	—
Body weight (kg)	57.7 (51.2–69.8)	63.8 ± 11.6*	79.2 ± 16.6*
Body mass index	22.1 (19.6–25.0)	24.4 ± 3.4*	28.1 ± 4.6*
Body surface area (DuBois, m^2^)	1.64 (1.48–1.79)	—	—
Laboratory data			
Aspartate aminotransferase (IU/L)	21.0 (17.8–24.5)	—	—
Alanine aminotransferase (IU/L)	14.0 (11.0–22.0)	—	—
Alkaline phosphatase (IU/L)	241 (194–283)	—	—
Total bilirubin (mg/dL)	0.50 (0.40–0.62)	—	—
γ-glutamyl transpeptidase (IU/L)	28.0 (20.0–52.8)	—	—
Creatinine (mg/dL)	0.82 (0.69–0.92)	—	—
Krebs von den Lungen-6 (U/mL)	1067 (694–1560)	—	—
Surfactant protein D (ng/dL)	294 (182–409)	—	—
Pulmonary function test			
forced vital capacity (L)	1.90 (1.41–2.35)	2.42 ± 0.67*	2.71 ± 0.76*
% forced vital capacity (%)	62.3 (50.1–72.1)	80.9 ± 16.6*	79.7 ± 17.6*
% DLCO (%)	50.8 (41.3–63.6)	44.6 ± 11.4*	47.4 ± 13.5*
Six minute walk test			
lowest SpO_2_ (%)	82.0 (76.0–87.0)	—	—
walking distance (meter)	410 (340–480)	—	—
Concomitant therapy			
Prednisolone (%)	11 (16.2%)	9 (11.8%)	136 (21.3%)
Cyclosporine (%)	2 (2.9%)	0	0
Cyclophosphamide (%)	2 (2.9%)	0	0
Tacrolimus (%)	5 (7.4%)	0	0
Pirfenidone (%)	2 (2.9%)	0	0

Categorical data are presented as numbers (percentages) and continuous data are presented as medians (interquartile ranges). *Continuous data in INPULSIS trials are presented as the mean ± standard deviation. Abbreviations: DLCO = diffusing capacity for lung carbon monoxide.

### Hepatotoxicity of nintedanib

Adverse events in terms of hepatotoxicity are summarized in Table 2. A high proportion of patients had elevated levels of liver enzymes: 46 patients (67.6%) exhibited AST and/or ALT elevation and 16 patients (23.5%) were CTCAE grade ≥2 with a median interval of 6 days. Three patients experienced acute hypochondriac pain before AST and/or ALT elevation with a CTCAE grade ≥2 were detected. An elevation in γ-GTP was also frequently observed and CTCAE grade ≥2 appeared in 40 patients (58.8%).

**Table 2:** Hepatotoxicity.

	CTCAE grading of the worst value		
	All grade	≥2	≥3
Our patients (N = 68)			
AST elevation	44 (64.7%)	14 (20.6%)	6 (8.8%)
ALT elevation	38 (55.9%)	12 (17.6%)	2 (2.9%)
AST and/or ALT elevation	46 (67.6%)	16 (23.5%)	6 (8.8%)
ALP elevation	29 (42.6%)	1 (1.5%)	0
Total bilirubin elevation	5 (7.4%)	3 (4.4%)	2 (2.9%)
γ-GTP elevation	40 (58.8%)	17 (25.0%)	2 (2.9%)
**Nintedanib group in INPULSIS trials**			
Japanese patients (N = 76)			
AST elevation	26/72* (36.1%)	4 (5.3%)	2 (2.6%)
ALT elevation	30/71* (42.3%)	4 (5.3%)	1 (1.3%)
AST and/or ALT elevation	—	5 (6.6%)	3 (3.9%)
Overall population (N = 638)			
AST elevation	134/625* (21.4%)	21 (3.3%)	8 (1.3%)
ALT elevation	169/620* (27.3%)	28 (4.4%)	10 (1.6%)
AST and/or ALT elevation	—	32 (5.0%)	14 (2.2%)

Categorical data are presented as numbers (percentages). *The number of patients whose test results increased >the upper limit of the normal range/the number of patients whose test results were within the reference values at baseline. Abbreviations; AST = aspartate aminotransferase; ALT = alanine aminotransferase; ALP = alkaline phosphatase; T-Bil = total bilirubin; γ-GTP = γ-glutamyl transpeptidase; CTCAE = Common Terminology Criteria for Adverse Events.

### Comparison of characteristics and examination findings with and without hepatotoxicity

A comparison between a group of 16 patients who demonstrated AST and/or ALT elevation with a CTCAE grade ≥2 and another group of 52 patients is shown in Table 3. The BSA was significantly lower in the CTCAE grade ≥2 group than in the CTCAE grade <2 group (*p* = 0.0484). Body weight was also lower in CTCAE grade ≥2 group, although this difference did not reach statistical significance (*p* = 0.0765). No significant differences were observed in terms of age, gender, laboratory data at the first visit, pulmonary function tests, six minute walk test, and concomitant therapy between the 2 groups.

**Table 3:** Comparison of characteristics and examination findings with and without hepatotoxicity.

	hepatotoxicity	no hepatotoxicity	*p*-value
**Number of patients**	16	52	
**Age**	75.0 (68.8–76.3)	72.0 (68.0–76.0)	0.519
**Gender (male/female)**	11/5	41/11	0.502
Physique			
Height (cm)	160 (153–166)	164 (158–169)	0.188
Body weight (kg)	53.2 (49.2–57.9)	62.0 (52.3–70.3)	0.0765
Body mass index	21.5 (19.4–24.3)	22.4 (20.0–25.0)	0.311
Body surface area (DuBois, m^2^)	1.52 (1.45–1.67)	1.67 (1.54–1.80)	0.0484
Laboratory data			
Aspartate aminotransferase (IU/L)	20.5 (16.5–31.5)	21.0 (18.0–24.0)	0.612
Alanine aminotransferase (IU/L)	14.0 (11.0–29.0)	14.0 (11.0–20.3)	0.524
Alkaline phosphatase (IU/L)	247 (211–280)	240 (189–283)	0.608
Total bilirubin (mg/dL)	0.50 (0.40–0.62)	0.50 (0.40–0.62)	1.00
γ-glutamyl transpeptidase (IU/L)	28.5 (21.5–63.8)	28.0 (20.0–48.0)	0.573
Creatinine (mg/dL)	0.80 (0.68–0.90)	0.83 (0.70–0.92)	0.385
Krebs von den Lungen-6 (U/mL)	920 (694–1356)	1080 (766–1592)	0.800
Surfactant protein D (ng/dL)	262 (167–440)	309 (188–400)	0.691
Pulmonary function test			
forced vital capacity (L)	1.75 (1.37–2.42)	1.96 (1.65–2.34)	0.457
%forced vital capacity (%)	64.0 (55.0–71.7)	60.9 (48.9–72.7)	0.822
%DLCO (%)	51.4 (36.1–60.1)	50.4 (42.2–65.6)	0.464
Six-minute walk test			
lowest SpO_2_ (%)	81.5 (77.5–87.0)	82.0 (76.0–87.0)	0.922
walking distance (meter)	378 (281–450)	430 (355–480)	0.299
Concomitant therapy			
Prednisolone (%)	3 (18.8%)	8 (15.4%)	0.712
Cyclosporine (%)	0	2 (3.8%)	1.00
Cyclophosphamide (%)	0	2 (3.8%)	1.00
Tacrolimus (%)	1 (6.2%)	4 (7.7%)	1.00
Pirfenidone (%)	1 (6.2%)	1 (1.9%)	0.418

Categorical data are presented as numbers (percentages) while continuous data are presented as medians (interquartile ranges). Fisher’s exact test was used to compare categorical data and the Mann-Whitney U test was used to compare continuous data. Abbreviations: DLCO = diffusing capacity for lung carbon monoxide.

### Risk factors for hepatotoxicity of nintedanib

We evaluated the risk factors for AST and/or ALT elevation with a CTCAE grade ≥2 (Table 4). Referring to the results of comparison with and without hepatotoxicity, we selected BSA not only as the most possible candidate risk factor, but also as a representative factor related to physique. We also selected age, %FVC, %DLCO (these 3 factors had a significant impact on the assessment of severity and/or prognosis of IPF and have little relation with physique), and the baseline AST and ALT levels as candidate risk factors. A multivariate logistic regression analysis showed that the association between BSA and AST and/or ALT elevation with a CTCAE grade ≥2 was statistically significant (*p* = 0.0457).

**Table 4:** Multivariate logistic regression analysis.

	Odds ratio	95% confidence interval	*p*-value
Body surface area	0.0208	0.000468–0.929	0.0457
Age	1.01	0.888–1.15	0.891
% forced vital capacity	0.988	0.947–1.03	0.564
% diffusing capacity for lung carbon monoxide	0.999	0.961–1.04	0.950
Baseline aspartate aminotransferase	1.09	0.950–1.25	0.224
Baseline alanine aminotransferase	1.00	0.932–1.08	0.994

**Figure 1:**
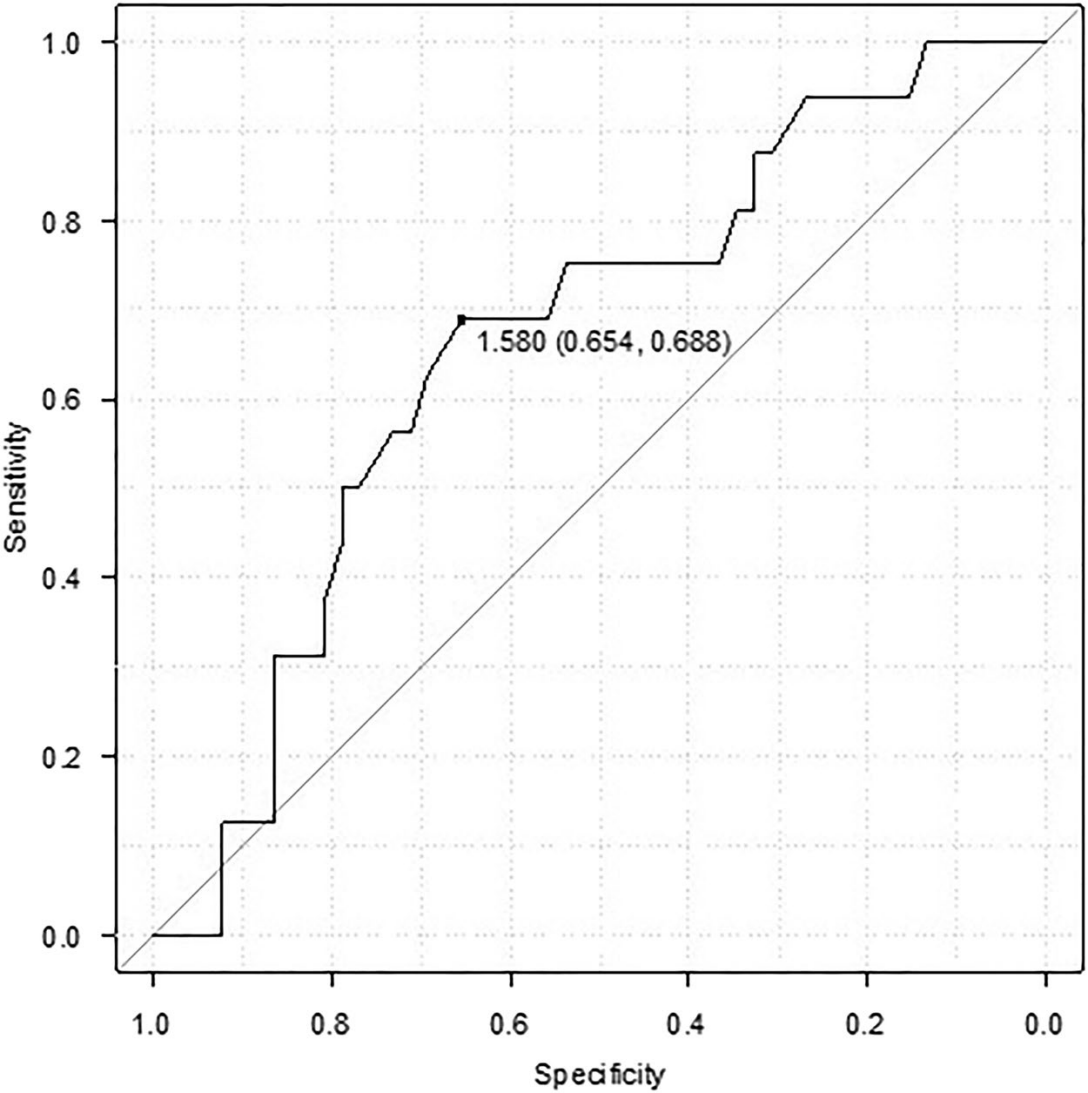
Receiver operating characteristic curve analysis. A receiver operating characteristic curve analysis was used to determine the cut-off values of body surface area. The area under the curve was 0.664 (95% confidence interval: 0.515–0.813) and the cut-off value for which sensitivity + specificity is maximal was 1.58 m^2^ (68.8% sensitivity and 65.4% specificity).

A ROC curve analysis was used to determine the BSA cut-off values (Fig. 1). The area under the curve for BSA was 0.664 (95% confidence interval: 0.515–0.813) and the cut-off value for which sensitivity + specificity is maximal was 1.58 m^2^ (68.8% sensitivity and 65.4% specificity).

### Treatment after interruption due to hepatotoxicity

Treatments after interruption due to hepatotoxicity are summarized in Table 5. In 16 patients, treatment interruption was required due to AST and/or ALT elevation with a CTCAE grade ≥2. In all cases, hepatic enzyme elevations were completely reversible with treatment interruption. Among 16 patients who needed a treatment interruption due to AST and/or ALT elevation with a CTCAE grade ≥2, re-administration at a reduced dose of 100 mg twice daily was performed in 10 patients. Treatment was successfully continued in 6 patients, whereas it was stopped in 4 patients due to the recurrence of AST and/or ALT elevation with a CTCAE grade ≥2 (2 patients) and nausea or fever (1 patient each). On the other hands, the remaining 6 patients who needed a treatment interruption due to AST and/or ALT elevation with a CTCAE grade ≥2 discontinued nintedanib treatment without resumption because of patient rejection or acute hypochondriac pain (2 patients each) and a deterioration in their physical condition or eosinophilia (1 patient each).

**Table 5:** Treatment after interruption due to AST and/or ALT elevation with a CTCAE grade ≥2.

	n
Resume at a reduced dose of 100 mg twice daily	10
Continue	6
Re-interrupt	4
recurrence of AST/ALT elevation with CTCAE grade ≥2	2
nausea	1
fever	1
Discontinue without resumption	6
patient rejection	2
acute hypochondriac pain	2
deterioration of physical condition	1
eosinophilia in peripheral blood	1

Abbreviations; AST = aspartate aminotransferase; ALT = alanine aminotransferase; CTCAE = Common Terminology Criteria for Adverse Events.

## Discussion

The present study demonstrated the 3 following important clinical observations. First, a low BSA was associated with AST and/or ALT elevation with a CTCAE grade ≥2 when treated with nintedanib at a dose of 150 mg twice daily. Second, 80% of the patients who resumed nintedanib at a reduced dose of 100 mg twice daily after treatment interruption due to hepatotoxicity did not again develop AST and/or ALT elevation with a CTCAE grade ≥2. Third, 37.5% of the patients who needed treatment interruption due to hepatotoxicity could not resume nintedanib treatment.

To date, risk factors for hepatotoxicity of nintedanib in patients with IPF have not been fully investigated. However, the present study suggested that a low BSA predicts an AST and/or ALT elevation with a CTCAE grade ≥2 when treating with nintedanib at a dose of 150 mg twice daily. The incidence of hepatotoxicity in the present study was considerably higher than that reported in the INPULSIS trials (as shown in Table 2), whereas factors related to physique such as body weight, BMI, and absolute FVC values were considerably lower than those reported in the INPULSIS trials (Table 1)^4^. Similarly, a sub-analysis of the INPULSIS trials revealed that the incidence of AST and/or ALT elevation was higher in Japanese populations than in overall populations (Table 2), whereas body weight, BMI, and absolute FVC values were lower in Japanese patients than those in the overall population (Table 1)^4, 5, 8, 9^. Moreover, in a phase I study of nintedanib combined with docetaxel in Japanese patients with advanced non-small-cell lung cancer, the incidence of hepatotoxicity was higher in patients with a BSA < 1.50 m^2^ than in patients with a BSA ≥1.50 m^2^ ^10^. These results indicate that physique is related to hepatotoxicity of nintedanib. Among the factors related to physique, BSA would be the most useful predictive factor.

It is also noteworthy that 8 of 10 patients (80%) successfully resumed nintedanib at a reduced dose of 100 mg twice daily after treatment interruption due to AST and/or ALT elevation with a CTCAE grade ≥2 at a dose of 150 mg twice daily. These 8 patients also had a small physique, with a median BSA of 1.57 m^2^. According to the pharmacokinetic analysis of nintedanib in Japanese patients, the area under the concentration-time curve and maximum concentration in plasma at a steady state were approximately two-times higher at a dose of 150 mg twice daily than those at a dose of 100 mg twice daily (39.7 ng/ml vs 20.0 ng/ml and 218 ng·h/ml vs 115 ng·h/ml, respectively)^11^. These data suggested that hepatotoxicity would be associated with the plasma concentration of nintedanib. In the present study, it was speculated that the patients with a small build tended to have a high serum concentration at a dose of 150 mg twice daily, and thus, were more likely to develop AST and/or ALT elevation. However, as the bioavailability of nintedanib is relatively low, serum concentration of nintedanib may differ among individuals. Therefore, further pharmacokinetic analysis in various patients with different physiques is required.

In the present study, an AST and/or ALT elevation was completely reversible with a treatment interruption. However, 6 of the 16 patients (37.5%) who needed a treatment interruption due to AST and/or ALT elevation with a CTCAE grade ≥2 could not resume nintedanib treatment. It might have been unavoidable that those patients could not resume because of concerns over noteworthy side-effects such as eosinophilia in the peripheral blood and acute hypochondriac pain. However, at least another 3 patients were highly likely to have continued nintedanib treatment over a longer period if it were not for the interruption. In addition, recently, an interim analysis of the INPULSIS^®^-ON study showed that the beneficial effect of nintedanib on slowing disease progression was maintained and the change from baseline FVC was consistent over 2 or more years^12^. Thus, it is very important to continue nintedanib treatment as long as possible without interruption and/or discontinuation by setting the appropriate dosage for individual patients. For patients with a small physique, especially Japanese and eastern Asian patients with a BSA <1.58 m^2^, it would be a good option to start nintedanib at a dose of 100 mg twice daily and then increase the dose to 150 mg twice daily if possible after confirming its safety.

A limitation of the present study was the retrospective single-center study design. In addition, frequent blood sampling may provide an opportunity to detect a temporal AST and/or ALT elevation that may recover spontaneously. In the present study, the median interval from nintedanib initiation to AST and/or ALT elevation with a CTCAE grade ≥2 was only 6 days, whereas the protocol of the INPULSIS trials specified that hepatic enzymes must be examined once every 2 weeks during the first 6 weeks. The short observation period was also another limitation when assessing long-term safety.

In conclusion, a low BSA was associated with hepatotoxicity of nintedanib at a dose of 150 mg twice daily in patients with IPF. To continue nintedanib treatment as long as possible without interruption and/or discontinuation, it would be a good option for patients with a small physique to start nintedanib at a dose of 100 mg twice daily and then increase the dose to 150 mg twice daily if possible after confirming its safety.

## Methods

### Patients and settings

This retrospective study was performed at the Kanagawa Cardiovascular and Respiratory Center in Yokohama City, Kanagawa, Japan. All consecutive cases of IPF newly treated with nintedanib at a dose of 150 mg twice daily from September 2015 to September 2016 were enrolled. The diagnosis of IPF was based on the official American Thoracic Society/European Respiratory Society/Japanese Respiratory Society/Latin American Thoracic Association statement of 2011^13^. Patients with a previous history of nintedanib treatment (formerly known as BIBF1120) were excluded. This study has been carried out in accordance with the Declaration of Helsinki. The Ethics Committee of the Kanagawa Cardiovascular and Respiratory Center approved the study protocol (Approval date: November 30, 2016; Approved number: KCRC-16-0006) and patient consent was waived because this was a retrospective study and anonymity was secured.

### Data availability

The datasets generated during and/or analysed during the current study are available from the corresponding author on reasonable request.

### Clinical and laboratory findings

Clinical and laboratory data used in this study were retrieved from patient medical records and included age, gender, height, body weight, laboratory data [AST, ALT, alkaline phosphatase (ALP), total bilirubin (T-Bil), γ-glutamyl transpeptidase (γ-GTP), serum creatinine, Krebs von den Lungen-6 (KL-6), and surfactant protein D (SP-D)], pulmonary function tests, six minute walk test, and concomitant therapy. In all cases, hepatic enzymes were examined at least once within a week of nintedanib treatment initiation and at least once every 2–4 weeks thereafter.

### Assessment and response for the hepatotoxicity

The worst examination values were graded using the CTCAE ver. 4.0^14^. An elevation in AST and ALT was defined as follows: grade 1 was >3.0 × the upper limit of the normal range (ULN), grade 2 was >3.0–5.0 × ULN, grade 3 was >5.0–20.0 × ULN, and grade 4 was >20.0 × ULN. When a patient developed AST and/or ALT elevation with CTCAE grade ≥2, treatment interruption or dose reduction was needed in accordance with the guide for appropriate use of nintedanib (Ofev^®^)^15^.

### Statistical analysis

Categorical data are presented as numbers (percentages) while continuous data are presented as medians (interquartile ranges). Fisher’s exact test was used to compare categorical data and the Mann-Whitney U test was used to compare continuous data. A multivariate logistic regression analysis was performed to verify the risk of hepatotoxicity. A receiver operating characteristic (ROC) curve analysis was used to determine the optimal cut-off values for the risk factor; values with maximum joint sensitivity and specificity were selected. A *p*-value of <0.05 was considered statistically significant. All statistical analyses were performed with EZR (Saitama Medical Center, Jichi Medical University, Saitama, Japan)^16^, which is a graphical user interface for R Version 3.2.2 (The R Foundation for Statistical Computing, Vienna, Austria).
